# INTERVENTION DEVELOPMENT USING PALLIATIVE CARE CLINICAL PRACTICE GUIDELINES CONCERNING SPIRITUALITY

**DOI:** 10.1093/geroni/igad104.0929

**Published:** 2023-12-21

**Authors:** Monica Beck, Amelia Gallagher

**Affiliations:** University of Alabama in Huntsville, Huntsville, Alabama, United States; The University of Alabama in Huntsville, Huntsville, Alabama, United States

## Abstract

Nurses may be reticent to engage older adult cancer patients in spiritual histories despite evidence of improved health outcomes citing lack of education, skills, and time as barriers. Few interventional studies target these barriers using the Clinical Practice Guidelines for Quality Palliative Care as a framework for providing spiritual care excellence. Lift the Spirit, an enhanced educational communication intervention, integrated the guidelines for assessing and respecting patient and family spiritual beliefs, provider self-reflection, and spiritual screening and assessment using a validated instrument. The mixed-methods pilot study aimed to assess the feasibility and acceptability of Lift the Spirit. A purposive sample of nurses (n = 17) viewed an educational module, role-played conducting spiritual histories using the Faith, Importance, Community, Address (FICA) © instrument, and completed debriefing interviews. Pre/post outcome measures of Spiritual History Knowledge and Spiritual History Self-Efficacy were analyzed using a Wilcoxon paired signed-rank test. Debriefing interview data were content analyzed. The Theoretical Framework of Acceptability was used to content code and identify acceptability-related content. There were significant differences between pre and post knowledge and self-efficacy scores, respectively (Wilcoxon Signed-Ranks: Z = -3.18, p = 0.002; and Z = -3.20, p = 0.001). Qualitative findings supported the feasibility and acceptability of Lift the Spirit; participants found the FICA© instrument and role-playing especially helpful. Preliminary findings suggested Lift the Spirit was feasible, acceptable, and positively impacted knowledge, skills, and self-efficacy. The utility of an intervention like Lift the Spirit to improve older adult cancer patient outcomes was demonstrated.

